# Comparative Protein Interaction Network Analysis Identifies Shared and Distinct Functions for the Human ROCO Proteins

**DOI:** 10.1002/pmic.201700444

**Published:** 2018-04-17

**Authors:** James E. Tomkins, Sybille Dihanich, Alexandra Beilina, Raffaele Ferrari, Nicolò Ilacqua, Mark R. Cookson, Patrick A. Lewis, Claudia Manzoni

**Affiliations:** ^1^ School of Pharmacy University of Reading Whiteknights Campus Reading UK; ^2^ Department of Molecular Neuroscience UCL Institute of Neurology London UK; ^3^ Laboratory of Neurogenetics National Institute on Aging National Institutes of Health Bethesda USA; ^4^ Department of Biology University of Padova Padova Italy

**Keywords:** LRRK2, LRRK1, DAPK1, MASL1/MAFHAS1, protein networks, ROCO proteins, protein microarrays

## Abstract

Signal transduction cascades governed by kinases and GTPases are a critical component of the command and control of cellular processes, with the precise outcome partly determined by direct protein–protein interactions (PPIs). Here, we use the human ROCO proteins as a model for investigating PPI signaling events—taking advantage of the unique dual kinase/GTPase activities and scaffolding properties of these multidomain proteins. PPI networks are reported that encompass the human ROCO proteins, developed using two complementary approaches. First, using the recently developed weighted PPI network analysis (WPPINA) pipeline, a confidence‐weighted overview of validated ROCO protein interactors is obtained from peer‐reviewed literature. Second, novel ROCO PPIs are assessed experimentally via protein microarray screens. The networks derived from these orthologous approaches are compared to identify common elements within the ROCO protein interactome; functional enrichment analysis of this common core of the network identified stress response and cell projection organization as shared functions within this protein family. Despite the presence of these commonalities, the results suggest that many unique interactors and therefore some specialized cellular roles have evolved for different members of the ROCO proteins. Overall, this multi‐approach strategy to increase the resolution of protein interaction networks represents a prototype for the utility of PPI data integration in understanding signaling biology.

## 1 Introduction

The subcellular environment hosts a dynamic network of molecular events that regulates cell homeostasis and coordinates signal transduction. Defining these regulatory mechanisms and understanding how they influence the physiology of biological processes is important in determining how subtle alterations in protein function may lead to disease. Since protein–protein interactions (PPIs) are central to these processes, and as interacting proteins are likely to be involved in the same or related pathway,^1^ searching for proteins that physically interact with each other represents a means to achieve deeper insight into the highly interconnected landscape of cellular functions. The importance of elucidating protein interactors within cell signaling events is illustrated in our understanding of the mTOR complexes,^2^ whereby the assembly of particular protein interactors differentially initiates a diverse range of functional pathways.

**Significance Statement**

This research demonstrates the utility of the extensive collection of PPI data already in the public domain via peer‐reviewed publication to complement novel PPI datasets, in order to identify similarities and differences in PPI and functional profiles of related multidomain proteins and prioritize interactors to pursue for validation in the laboratory. The human ROCO proteins are an attractive protein family for utilizing this approach since their primary structure consists of a conserved region flanked by a diverse range of PPI domains within a single open reading frame.

Our literature mining pipeline implemented in this analysis, WPPINA, ensures a wide coverage of reported PPIs from multiple data repositories which maximizes the usefulness of novel data integration, such as protein microarray as is used in this study. The significance of this strategy is that novel datasets are not just stand‐alone results and can be interpreted in combination with decades of research into PPIs of particular proteins of interest, by adopting this straightforward approach to support further investigations.

**Figure 1:**
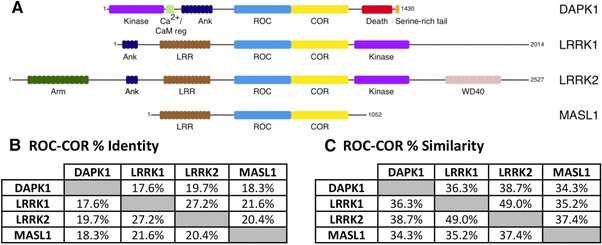
Domain topology of the human ROCO proteins and ROC‐COR supra‐domain sequence similarity. A) Multidomain structure of the human ROCO proteins which are characterized by a conserved tandem ROC‐COR domain. Abbreviations: Ank, ankyrin repeats; Arm, armadillo repeats; Ca^2+^/CaM reg, calcium/calmodulin regulatory domain; COR, C‐terminal of ROC; DAPK1, death‐associated protein kinase 1; LRR, leucine‐rich repeats; LRRK1, leucine‐rich repeat kinase 1; LRRK2, leucine‐rich repeat kinase 2; MASL1, malignant fibrous histiocytoma amplified sequence 1; ROC, Ras of complex proteins. B) Peptide sequence identity and C) similarity of the ROC‐COR supra‐domain across ROCO proteins. ROC‐COR region defined as residues 612–1225 (DAPK1), 574–1143 (LRRK1), 1271–1790 (LRRK2) and 345–972 (MASL1). Please note that the presence of a WD40 domain in LRRK1 is still a matter of scientific debate.^50^, ^52^

The human ROCO protein family (Figure 1) consists of four multidomain cell signaling proteins, death‐associated protein kinase 1 (DAPK1), leucine‐rich repeat kinase 1 (LRRK1), leucine‐rich repeat kinase 2 (LRRK2), and malignant fibrous histiocytoma amplified sequence 1 (MASL1 or MFHAS1), which are characterized by a tandem ROC (Ras of complex proteins)‐COR (C‐terminal of ROC) supra‐domain.^3^ Although the ROCO proteins are defined by this conserved region, the domain topology surrounding the ROC‐COR unit (which includes numerous protein interaction motifs) is diverse and dissimilar between ROCO proteins. Three of the four ROCO proteins (DAPK1, LRRK1, and LRRK2) also harbor active kinase domains in addition to the GTPase activity of the ROC domain, an arrangement that is exclusive to these three proteins, within the human proteome. The combination of multiple enzymatic activities coupled with a range of PPI domains within the same open reading frame positions the ROCO proteins as a unique protein family to investigate the functional commonalities and differences of structurally related proteins. The presence of several interaction domains within the primary structure of these proteins may reduce the requirement for adaptor proteins to complex with ROCO proteins. Therefore, direct interactors are likely to be functionally relevant effector proteins and hence the analysis of direct ROCO protein interactors will provide important functional insight into this family of proteins. Thus, the human ROCO proteins are an attractive protein family to utilize as a model for PPI network analysis, to explore the link between PPI profiles and functional fates. This approach has been previously used for LRRK2 in isolation,^4^, ^5^ the DAPK1 interactome has been reviewed,^6^ and the comparison between LRRK1 and LRRK2 has been attempted.^7^ However, the collective PPI network analysis of the entire human ROCO protein family is a novel contribution.

Despite their sequence similarity (Figure 1), the human ROCO proteins appear to be associated with different cellular processes. DAPK1 is linked to cell death pathways^8^, ^9^, ^10^ and is also involved in inflammation.^11^ LRRK1 has been associated with numerous distinct cellular mechanisms, which include EGFR trafficking,^12^ mitotic spindle orientation,^13^ and humoral immunity.^14^ LRRK2 has been implicated in a diverse range of cellular processes, including macroautophagy, cytoskeletal dynamics, and mitochondrial function.^15^ Finally MASL1, the least studied of the human ROCO proteins, has functional connections to macrophage polarization^16^, ^17^ and erythropoiesis.^18^ These proteins also have disease relevance: DAPK1, LRRK1, and MASL1 in cancers,^19^, ^20^, ^21^ while mutations in LRRK2 are a common genetic contributor to familial Parkinson's Disease (PD)^22^ and LRRK2 has been associated with numerous other human diseases.^23^ However, significant gaps in our understanding of ROCO protein biology persist, which have implications for drug development in human disease.^19^, ^24^ In addition, fundamental questions relating to why such similar proteins are differentially involved in health and disease, and how the complex enzymatic functions of these proteins fit with the biochemical regulation of cellular signaling pathways, remain to be addressed.

Since key components underlying the functional divergence evident between the ROCO proteins will reside in their proximal interactomes,^7^, ^23^ we set out to investigate these interactomes using two orthologous approaches to determine PPI networks across the human ROCO protein family. We first used an in‐house data mining approach which enabled identification of PPIs reported in the published literature, to generate a weighted protein–protein interaction network analysis (WPPINA).^25^ Second, to complement WPPINA, we used protein microarray screens to construct an experimental network, enabling hypothesis‐free discovery of novel protein interactors. We compared these two ROCO protein networks to validate interactors across the approaches and prioritize interactors for further investigation. Functional insight into these networks was obtained by utilizing gene ontology (GO) functional annotations. These results highlight a subset of interactors common to multiple ROCO proteins, but also numerous interactors specific to particular ROCO proteins, supporting the hypothesis that these proteins have evolved largely independent cellular functions.

Furthermore, we demonstrate that the use of WPPINA to query a high‐throughput‐derived PPI dataset (such as data obtained by protein microarray screens) represents a novel, rapid, and effective tool to prioritize protein interactors for further experimental validation based on the functional knowledge that is readily available in the published literature.

## 2 Experimental Section

### 2.1 Literature‐Derived Network Data Download

Protein–protein interaction data was obtained by querying the PSICQUIC online interface^26^ (available at http://www.ebi.ac.uk/Tools/webservices/psicquic/view/main.xhtml) for DAPK1, LRRK1, LRRK2, and MASL1, independently. Data was downloaded on January 12, 2017, in a MITAB 2.5 format, from six primary database sources: IntAct,^27^ BioGRID,^28^ InnateDB, Innate‐DB‐All, InnateDB‐IMEx,^29^ and MINT^30^ to ensure a wide capture of reported PPIs.

### 2.2 Construction of the Literature‐Derived Network

The literature‐derived ROCO PPI network was constructed as previously described.^25^ In brief, datasets from primary PPI databases were processed to obtain format and protein identifier (ID) consistency, utilizing a dictionary dataset of all human proteins (developed from a UniProt search of human proteins obtained on January 13, 2017). Data from the six datasets were then merged into a single file and repeated equivalent interaction data entries (i.e., interactions derived from the same publication and annotated in multiple databases) were removed.

A series of filtering steps were applied. First, non‐protein interactors, such as chemical and miRNA, and protein ID terms corresponding to non‐reviewed automatic annotations, which include UniProt TrEMBL IDs, were removed. In addition, transcript‐specific information was removed. Next, non‐human interactors, which included seed orthologs, were discarded. Filtered datasets were then subjected to method detection reassignment, which grouped similar detection methods based on the EBI Molecular Interactions Ontology, available at http://www.ebi.ac.uk/ols/ontologies/mi (File 1, Supporting Information).

A confidence value was assigned to each interaction based on three parameters: method score (MS), the number of different methods used to detect a specific interaction (one method scored a value of 1, multiple methods scored a value of 2); publication score (PS), the number of publications that report a specific interaction (one publication scored a value of 1, multiple publications scored a value of 2); and CRAPome score (CS), the likelihood that the interaction is an affinity purification mass spectrometry (APMS) contaminant. The CS utilizes the CRAPome^31^ (version 1.1), a known contaminant repository for APMS experiments, which contained 411 datasets at the time of scoring (January 18, 2017). Each interactor that was detected by APMS was queried against the CRAPome and if the protein was a positive hit in >50% of the CRAPome datasets and had only been detected by APMS, the protein was scored a value of −1; if the protein was a positive hit in >50% of the CRAPome datasets but had also been detected by another non‐APMS method or was a positive hit in 30–50% of the CRAPome dataset and had only been detected by APMS, the protein was scored a value of −0.5; and if the protein was a positive hit for <50% of the CRAPome datasets and had also been detected by another non‐APMS method or was a positive hit in <30% of the CRAPome datasets, the protein was scored a value of 0.

The sum of the three scoring parameters then formed the basis of a confidence scale and only interactions that scored <2 were retained for constructing the network. This <2 score threshold ensures that nodes of the network represent interactors that have been independently replicated, by method and/or publication.

### 2.3 Protein Production and Purification

HEK293T cells were transfected with 3xFLAG tagged DAPK1, LRRK1, LRRK2, MASL1, or GFP plasmids using PEI reagent, collected 24 h after transfection and cells were lysed in the buffer: 20 mM Tris (pH 7.5), 150 mM NaCl, 1 mM EDTA, 1% Triton, 10% Glycerol, protease inhibitor cocktail (Roche), and 1x Halt phosphatase inhibitor cocktail (Thermo Scientific). Lysates were precleared by centrifugation at 20 000 × *g* for 10 min and incubated for 1 h at 4 °C with EZview Red Protein G beads (Sigma) to remove proteins non‐specifically binding to agarose. After preclear with protein G beads, lysates were incubated for 1 h at 4 °C with EZview Red Anti‐FLAG M2 Agarose (Sigma) that is suitable for the immunoprecipitation of FLAG fusion proteins. Beads were washed six times with the wash buffer: 20 mM Tris (pH 7.5), 400 mM NaCl, 1% Triton and proteins were eluted in 25 mM Tris (pH 7.5), 150 mM NaCl, and 100 μg mL^−1^ 3xFLAG peptide (Sigma). Protein yields and purity were estimated by staining gels with Coomassie brilliant blue staining (Thermo Scientific, Figure 1, Supporting Information).

### 2.4 Protein Microarrays

3xFLAG tagged, full‐length DAPK1, LRRK1, LRRK2, MASL1, and GFP control proteins were purified as previously described.^32^ Six micrograms of each purified 3xFLAG tagged protein were used to probe protein microarrays (Protoarray, version 4.1; Invitrogen) according to the manufacturer's instructions with the modification that after 3xFLAG tagged protein probing, arrays were probed with monoclonal ANTI‐FLAG BioM2−Biotin, Clone M2 (Sigma‐Aldrich) antibody, followed by probing with Alexa Fluor 647 streptavidin (Invitrogen).^33^ Arrays were imaged using an Axon GenePix 4000B fluorescence scanner and images were analyzed using GenePix Pro software. ProtoArray Prospector software was used to analyze the microarray data acquired from GenePix Pro and identify the significant hits. Binding strength was estimated as Z‐scores, that is, numbers of standard deviations above background fluorescence on the array. Each protein on the array was spotted in duplicate, hence reported values were averaged for both spots. Signals considered as potential interactions were determining using a Z‐score threshold of Z > 3. ROCO protein positive hit interactors were determined by filtering against GFP (negative control) interactions to identify proteins that bound DAPK1, LRRK1, LRRK2, or MASL1 but not GFP.

### 2.5 Functional Annotation

To gather insight into the cellular processes that are influenced by the proteins within the networks, functional enrichment analysis was performed. This analysis is based on gene ontology (GO) annotations and determines enrichment of biological process (BP) annotations within a query protein list (ROCO protein interactors in this case), by a comparison against annotations for the entire human genome. Functional enrichment analysis was undertaken using g:Profiler g:GOSt (available at http://biit.cs.ut.ee/gprofiler/index.cgi), on June 23, 2017. Statistical significance was determined using Fisher's one‐tailed test with a g:Profiler g:SCS algorithm to correct for multiple testing; *p* < 0.05 was set as the significance threshold and output data was not subjected to hierarchical filtering. Results were confirmed by replication of the functional enrichment analysis using WebGestalt^34^ (http://webgestalt.org/option.php) and Panther^35^ (http://www.pantherdb.org/) on November 22, 2017 (File 10, Supporting Information); the statistical testing underlying the enrichment analysis for these alternative portals is different, thus replication by this means provides reinforcement of the result obtained using g:Profiler.

All algorithms used for data processing were developed in R version 3.2.2. Networks were generated and visualized using Cytoscape^36^ version 3.3.0 and graphs were produced in GraphPad Prism 7.0.

## 3 Results

We here present an insight into the protein interaction network of the ROCO protein family. The four human ROCO proteins, DAPK1, LRRK1, LRRK2, and MASL1 were used as seed proteins. The term “interactome” refers to the group of proteins that directly bind to a particular seed protein.

### 3.1 Construction of the Literature‐Derived Network

**Figure 2:**
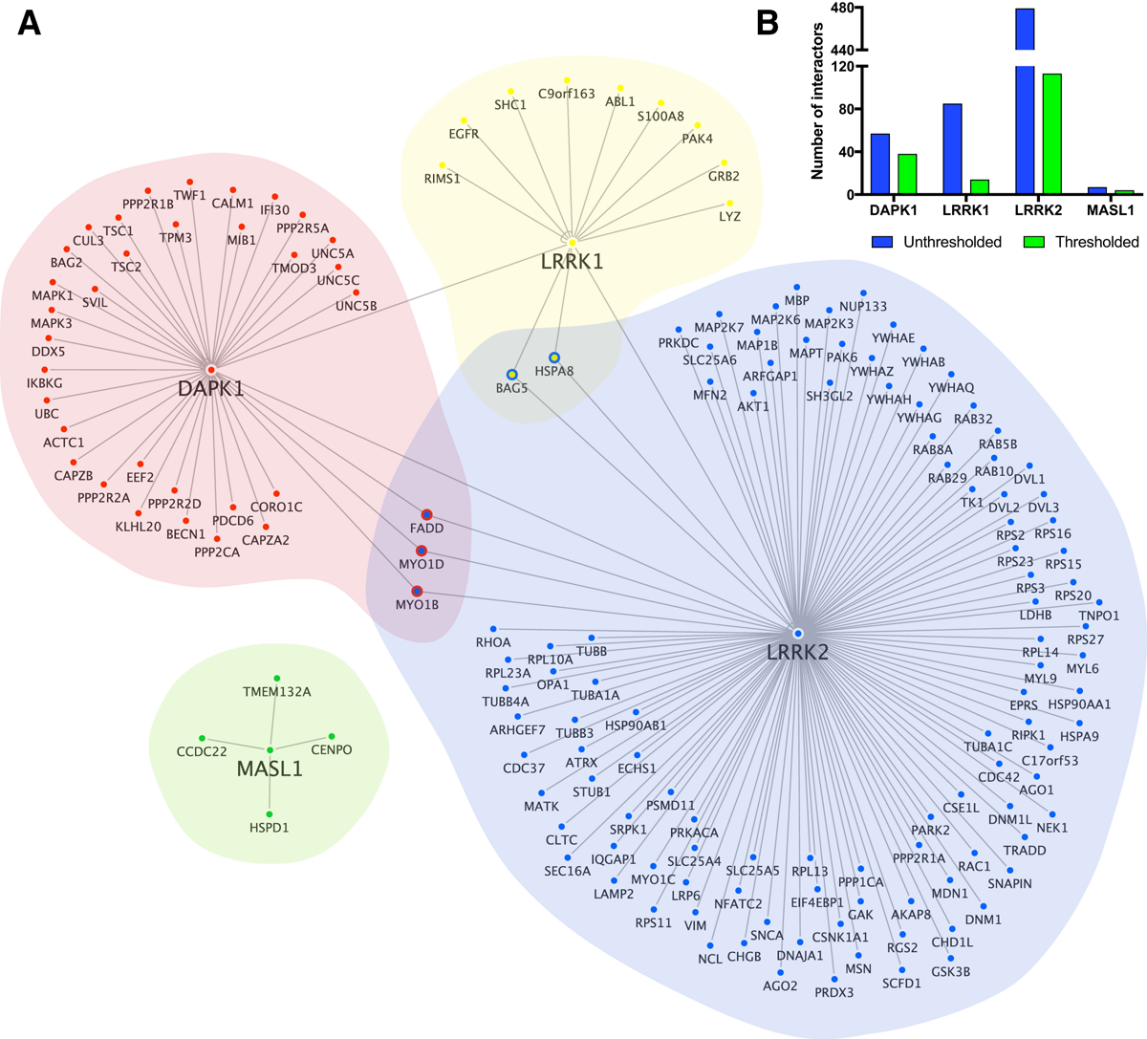
Literature‐derived ROCO protein interaction network. A) Network visualization of the ROCO protein interaction partners following data processing via the WPPINA pipeline. B) Quantification of the interactors retained following confidence score thresholding.

The literature‐derived PPI network (Figure 2A) was constructed by collecting the reported PPIs of ROCO proteins, utilizing our recently developed pipeline (WPPINA),^25^ which collates data from several databases within the IMEx consortium.^37^ Data were quality checked and a confidence threshold was applied to retain only interactions that have been replicated by a minimum of two experimental methods and/or reported in at least two peer‐reviewed publications. Therefore, this network provides a confidence‐weighted visual overview of state‐of‐the‐art PPI knowledge centered on the human ROCO proteins.

The network topology indicated a strong bias toward the LRRK2 interactome with 113 interactors, compared to the 38, 14, and 4 interactors for DAPK1, LRRK1, and MASL1, respectively (Figure 2B). This differential recovery of PPIs is likely driven by literature bias toward proteins with known human disease associations. For example, LRRK2 is the focus of many investigations within PD research,^38^ whereas MASL1 is relatively understudied.^21^ Interestingly, this trend differs when considering the interactomes prior to applying the confidence threshold (i.e., when retaining all reported interactors regardless of replication; Figure 2B). Of the 57 DAPK1 interactors reported within the literature, 38 were retained when the confidence threshold was applied. This relatively high (66.7%) retention of interactors indicates that the majority of interactors that have been identified for DAPK1 have been replicated. Four of the seven (57.1%) reported MASL1 interactors were also replicated observations and hence were retained for constructing the network. In contrast, only 16.5% of LRRK1 interactors and 23.5% of LRRK2 interactors were retained after confidence thresholding, showing limiting replication of the interactors identified. These results suggested that the expanse of PPI data for more widely studied proteins does not directly reflect increased confidence or robustness of the related interactome.

Considering only the confidence thresholded network, our results indicated common interactors between ROCO proteins: three interactors common to both DAPK1 and LRRK2 (FADD, MYO1B, and MYO1D), and two interactors common to both LRRK1 and LRRK2 (BAG5 and HSPA8; Figure 2A). Functional insight into these common interactors is summarized in Table 1, Supporting Information. In addition, from this analysis it was shown that DAPK1, LRRK1, and LRRK2 can exist as homo‐ and hetero‐dimers, conformations that may be critical for the functions of these proteins.^39^, ^40^ In contrast, the MASL1 interactome was fully detached from the other ROCO protein interactomes within this network, indicating a lack of common interactors between MASL1 and the other ROCO proteins on the basis of the existing literature.

### 3.2 Generating the Experimental Network

**Figure 3:**
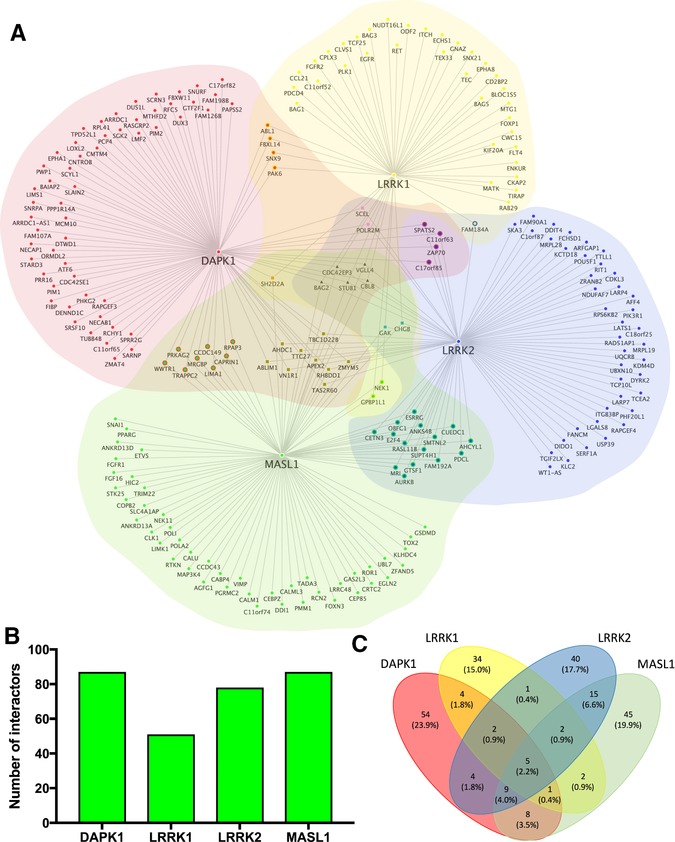
Experimental ROCO protein interaction network. A) ROCO protein interaction network analysis using protein microarray screens. B) Quantification of positive interactors identified by protein microarray for each ROCO protein. Three hundred and three interactions identified across 226 nodes. C) Extent of common nodes within the experimental network. Number of interactors and percentage of entire experimental network reported.

To address the biases in literature coverage for the human ROCO proteins, we performed protein microarray experiments as a hypothesis‐free approach for identifying potential ROCO protein interaction partners. This approach formed the basis of the experimental network (Figure 3A). We limited false‐positive hits in each interactome by setting a Z‐score threshold to distinguish positive hits from background signals and by filtering ROCO protein hits against GFP hits as a negative control for non‐specific binding.

In contrast to the literature‐derived network, this network displayed a more even distribution of interactors around each seed protein (Figure 3B). Specifically, we identified 87 (DAPK1), 51 (LRRK1), 78 (LRRK2), and 87 (MASL1) positive hits for each seed protein, respectively (File 2, Supporting Information). Of note, numerous kinases have been identified as potential MASL1 interactors (Table 1), six of which, CLK1, LIMK1, MAP3K4, NEK11, ROR1, and STK25, appear to be specific interacting partners of MASL1.

**Table 1:** MASL1‐interacting kinases. Kinases that were identified as interactors of MASL1 in the protein microarray screen, with functional associations. Of note, cell cycle–related functions appear to be a common functional theme

Kinase interactor		Additional seed interaction	Functional enrichment contribution	Further functional detail
Abbreviated name	Full protein name			
AURKB	Aurora kinase B	LRRK2	‐	Interacts with CLK1,^53^ another MASL1‐interacting kinase identified in this protein microarray screen Phosphorylated AURKB localizes to kinetochores in prometaphase cells^54^ Functional role in mitotic cell division, specifically as a catalytic unit of the chromosomal passenger complex (CPC)^54^ Dysregulation associated with tumorogenesis^55^
CLK1^a^	CDC2‐like kinase 1	‐	‐	Associates and phosphorylates AURKB,^53^ another MASL1‐interacting kinase identified within this protein microarray screen Dual specificity kinase that localizes to the nucleus^56^ Involved in alternative splicing and neuronal differentiation^56^, ^57^, ^58^ Potential drug target for Influenza and Alzheimer's disease (AD)^59^, ^60^
GAK	Cyclin‐G‐associated kinase	LRRK1 and LRRK2	Development, transport, intracellular organization, protein metabolism	Androgen receptor‐interacting transcriptional coactivator^61^ Localizes to the trans‐Golgi network^62^ Involved in clathrin‐mediated membrane trafficking and metaphase mitotic progression^63^ Disease links to cancer and Parkinson's disease (PD)^61^, ^64^, ^65^
LIMK1^a^	LIM domain kinase 1	‐	‐	Regulates microtubule dynamics, specifically mitotic spindle structure and positioning Acts downstream of several Rho‐family GTPase signal transduction pathways^66^
MAP3K4^a^	Mitogen‐activated protein kinase kinase kinase 4	‐	‐	Mediator in stress‐activated p38/MAPK and JNK signaling pathways^67^ Involved in tumur suppression and epithelial‐mesenchymal transition^68^ Loss of MAP3K4 is associated with defective neural tube development^69^
NEK1	NIMA‐related kinase 1	LRRK1	Cell Cycle, Intracellular Organization, Protein Metabolism, Response to Stimulus	Associated with axial spondylometaphyseal dysplasia^70^ Involved in DNA damage response and cell cycle control; suggested role in post‐mitotic cilia assembly Mutations in NEK1 are associated with ciliopathy and polycystic kidney disease (PKD)^71^
NEK11^a^	NIMA‐related kinase 11	‐	‐	Involved in DNA damage and genotoxic stress responses Highly expressed throughout S phase of the cell cycle to the G2‐M transition Activated by phosphorylation by ATM and ATR kinases^71^
ROR1^a^	Receptor tyrosine kinase‐like orphan receptor 1	‐	‐	Pseudokinase Non‐canonical Wnt transmembrane receptor^72^ Highly upregulated in chronic lymphocytic leukemia (CLL)^73^ and other blood cancers^74^
STK25^a^	Serine/threonine kinase 25	‐	‐	Associates with Golgi apparatus Dominant negative STK25 causes dispersal of the Golgi apparatus and inhibits cell migration^75^ Involved in glucose homeostasis^76^ Regulates lipid release from lipid droplets and induces NAFLD/NASH pathogenesis^77^

a kinases specific to MASL1

A remarkable finding from mapping this protein microarray data was that 23.5% of the entire network consisted of common node connections between two or more seed proteins. Furthermore, 8.4% of the nodes in the network were common to three or more seed proteins and five nodes (2.2% of the network) were common to all four seed proteins (Figure 3A,C). This suggested that the overlap between seed protein interactomes might in fact be greater than previously reported. However, it is important to note that further validation of these interactors is required to increase confidence in their veracity.

### 3.3 Identification of ROCO Protein Common Interactors

**Figure 4:**
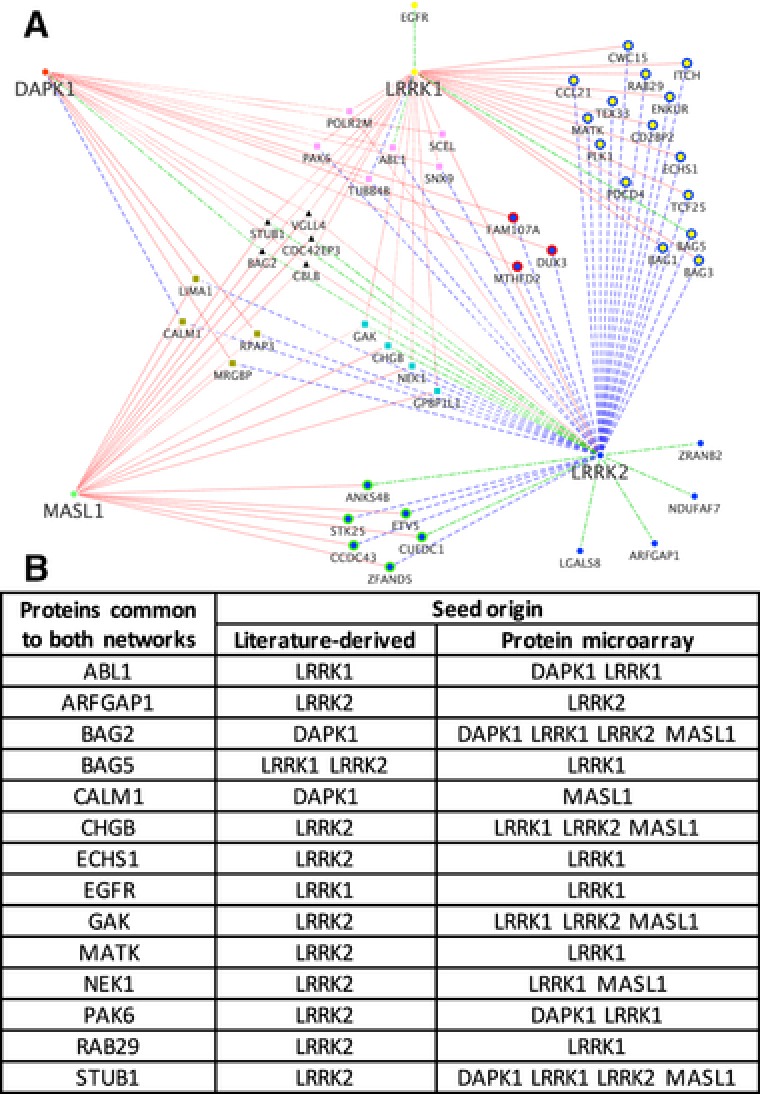
Common nodes across literature‐derived and experimental ROCO PPI data. A) The network is specifically depicted to highlight interactors that are common to both non‐thresholded literature‐derived data and protein microarray data. Dotted edges indicate interactions deriving from protein microarray experiments; dashed edges indicate interactions described in literature; dotted and dashed edges are interactions replicated between the two datasets. Seeds are represented with a circular node. Common interactors are represented with a double circular node if they are common to two seeds, square node if they are common to three seeds, and triangular node if they are common to all four seeds. B) Common nodes across the literature‐derived and experimental networks when considering literature‐derived data after thresholding.

A particular advantage of applying two orthologous network analysis approaches is to compare and combine the networks to minimize the burden of approach‐specific limitations and maximize the capacity of available data. To achieve this, we merged the literature‐derived network and the microarray data with the aim of validating via the literature some of the experimentally obtained, but not replicated, hits. Many nodes were common to both networks (Figure 4; referred to as the common core network). These common nodes can be categorized into three groups: i) interactors of the same seed protein that are cross‐supported by both networks (e.g., ARFGAP1, CHGB, and GAK which are common to LRRK2 in both networks); ii) interactors common to both literature‐derived data and the experimental network but within different seed protein interactomes (Figure 4), and iii) interactors that are common to both literature‐derived and experimental datasets associated with the same seed protein, but only if the confidence threshold is removed from the literature‐derived data (Table 2, Supporting Information). Interactors from iii) do not exceed the confidence threshold in place within the WPPINA pipeline to support replication of interactors, however with integration of the protein microarray data these interactors would exceed this threshold due to acquiring independent replication from the protein microarray experiments.

Considering the overlap between the literature‐derived and the experimental networks, 14 common interactors were identified (Figure 4B). When the non‐thresholded literature‐derived data and the protein microarray network were examined, 48 interactors were common to both datasets (Figure 4 and File 3, Supporting Information).

To further investigate the likelihood that the common core network reports true interactions, we added an additional score considering tissue‐specific gene expression. Using expression data derived from GTEx^41^ and a gene expression threshold of three reads per kilobase of transcript per million mapped reads (RPKM), co‐expression analysis identified distinct tissues where specific interactor mRNA were expressed together with specific seed protein mRNA (Table 3, Supporting Information).

Concerning pairwise interactions between ROCO proteins and interactors from the common core network (48 interactors and a total of 115 pairwise interactions; Table 3, Supporting Information), only 1 protein out of 48 interactors, DUX3, was not found in the GTEx database used for co‐expression analysis. On average, co‐expression within nine tissues was evident, whilst in ten cases co‐expression was found in 12 tissues (out of 13 analyzed, Table 3, Supporting Information). Although most tissues included at least one co‐expressed interaction pair, one tissue (skeletal muscle) did not show any co‐expressed interaction pairs due to an absence of significant ROCO protein expression in this tissue. The highest proportion of co‐expressed interaction pairs was seen in the reproductive apparatus (96% of co‐expressed interaction pairs), followed by two tissues: brain and intestine (92% and 90% of co‐expressed interaction pairs, respectively; Table 3, Supporting Information).

### 3.4 Functional Insight Into the Common Core Network

The literature‐derived and common core networks were subjected to functional enrichment analysis based on gene ontology (GO) functional annotations. Particularly, we used biological process (BP) terms to gather functional insight into these networks. The significantly enriched BP terms were grouped into functional blocks defined by more specific semantic classes (using a curated dictionary list to match GO terms with a custom grouped ontology) based on semantic similarity (Files 4 and 8, Supporting Information). This enabled an overview of significantly enriched functions (see Figure 2, Supporting Information for a summary of functions associated with the ROCO literature‐derived and common core networks, and Files 4–9, Supporting Information for a breakdown of each functional block, including semantic class– and GO term–specificity).

Within the common core network, which consists of 48 common interactors plus the seed protein nodes (Figure 4), a total of 26 GO BP terms were significantly enriched, representing a specific subset of the whole 516 functionally diverse terms significantly enriched within the literature‐derived network (Figure 2, Supporting Information and File 4, Supporting Information). The predominantly enriched terms within this refined analysis indicated “response to stimulus” and “intracellular organization” functional blocks supported by “stress” and “cell projections” semantic classes, respectively (Table 2). Functional associations for specific ROCO proteins were also explored by functional enrichment analysis of the individual interactomes within the literature‐derived network identifying “cell death” and “development” as distinct functional themes for DAPK1 and LRRK1, respectively, and “intracellular organization” and “transport” for LRRK2 (Table 2).

**Table 2:** Most significantly enriched terms from functional enrichment analysis of each dataset

Datasets	*p*‐value	GO term	Semantic class
Literature‐derived	4.6E‐36	Cellular component organization or biogenesis	Intracellular organization
Network	2.44E‐30	Intracellular transport	Transport—intracellular
	4.31E‐30	Cellular component organization	Intracellular organization
DAPK1	0.000000385	Cell death	Cell death
	0.000000439	Apoptotic process	Cell death—apoptosis
	0.00000136	Programmed cell death	Cell death
LRRK1	0.0000349	Neuron projection development	Development—neuronal—axon
	0.0000382	Cell development	Development
	0.0000911	Neurogenesis	Development—neuronal
LRRK2	2.75E‐29	Cellular component organization or biogenesis	Intracellular organization
	4.36E‐29	Intracellular transport	Transport—intracellular
	1.61E‐26	Establishment of localization in cell	Protein metabolism—localization
Common core	0.00000192	Regulation of cellular response to stress	Response to stimulus—stress
Network	0.0000539	Plasma membrane bounded cell projection organization	Intracellular organization—cell projections
	0.0000752	Cell projection organization	Intracellular organization—cell projections

## 4 Discussion

The human ROCO proteins are defined by a ROC‐COR supra‐domain which contains highly conserved motifs and substantial sequence similarity (Figure 1), a tandem domain organization that can be evolutionary traced from prokaryotic organisms.^42^ This domain homology is paralleled by flanking domain dissimilarity, driving a twofold interest into the proximal interactors of these proteins and their potential effects on subcellular functions: first from a fundamental biology perspective in relation to the complex domain organization of these proteins, and second from a drug discovery perspective due to the involvement of these proteins in human diseases.

In the current study, we used a combination of bioinformatic literature‐based analysis (WPPINA) and an experimentally derived protein microarray dataset, to expand our insight into the ROCO protein interactomes, specifically into common and distinct interactors, and functional pathways regulated by this family of proteins.

Although literature‐derived PPI networks are incomplete by definition as they are affected by ascertainment bias^43^ and depend solely on existing experimental findings (i.e., many interactors may exist that are yet to be discovered and/or relatively newly discovered protein interactors will be neglected in comparison to the more studied ones), the WPPINA analysis reported here represents a comprehensive literature review of reported ROCO PPIs^25^ and ensures an extensive and weighted coverage of primary literature sources comparatively to currently used literature mining and prediction network mapping tools.

In the case of the ROCO proteins, the literature‐derived PPI network incorporates a potential bias toward the LRRK2 interactome, due to extensive investigation into LRRK2 in relation to PD.^44^, ^45^ This is evidenced by the nearly twofold increase in the number of LRRK2 interactors (from 62 to 113) compared to a previous analysis performed in 2014 using an earlier version of the same data processing pipeline.^4^ Conversely, the distribution of nodes amongst the other ROCO proteins highlights the comparative neglect of research into characterizing the DAPK1, LRRK1, and MASL1 interactomes.^21^ However, it is worth considering that all interactions reported through WPPINA are experimentally proven, replicated, and cleared from type‐I error.

The domain topology and primary structures of the ROCO proteins are dissimilar outside of the ROC‐COR region (Figure 1), hence common interactors may provide hints toward ROC‐COR‐specific interactions. The common interactors identified within the literature‐derived network include: FADD, MYO1B, and MYO1D (between DAPK1 and LRRK2), and BAG5 and HSPA8 (between LRRK1 and LRRK2). Functional insight into these common interactors is provided in Table 1, Supporting Information. Of note, two common interactors (MYO1B and MYO1D) are unconventional myosin proteins involved in vesicle trafficking, a critical function for many cellular processes and ultimately cell survival. Interestingly, Rab proteins have a regulatory role in myosin motor function, which combined with evidence of Rab proteins as LRRK2 substrates^46^ and myosins as LRRK2 interactors, supports a key role for LRRK2 in the regulation of intracellular vesicle transport.^47^ This WPPINA approach allows for the straightforward identification of these mutual connections which could easily be overlooked when reviewing literature using alternative strategies. By removing the confidence threshold within the WPPINA pipeline, we increased the number of interactors reported within the literature‐derived ROCO PPI network; however, the additional interactors have to be considered carefully since there is no evidence of replication within the peer‐reviewed literature.

The experimental network, which is based on protein microarray data, provides novel insight into the ROCO protein interactomes. This network is not biased toward a specific seed protein since all are equally evaluated utilizing a hypothesis‐free approach and is complete in relation to the extensive range of proteins immobilized on the microarray (9480 proteins). However, the experimental network is not as robust as the literature‐derived network due to technical biases (i.e., intrinsic limitations to this experimental procedure, including the choice of baits for the microarray; alterations of physiological protein conformations [non‐physiological environment, absence of lipidic membranes, tagged preys]; variations of posttranslational modifications as evidence suggests that the phosphorylation state of LRRK2 impacts the protein interaction profile of the protein).^48^ Consequently, interactions reported in the experimental network require replication by alternative interaction detection methods to overcome the technical biases and ensuring validity of the protein microarray positive hits.

Nevertheless, this high‐throughput approach allows for the identification of potential novel interactors, expanding the current landscape of the ROCO protein interaction network, particularly for the less studied ROCO proteins. For example, many potential MASL1 interactors have been identified, which include numerous kinases (Table 1). MASL1 (unlike the other ROCO proteins) lacks an intrinsic kinase domain (Figure 1), therefore it can be hypothesized that its GTPase activity within the ROC domain may influence an extrinsic kinase domain.^39^, ^49^, ^50^ The novel potential MASL1‐interacting kinases identified in this screen may be downstream effectors of the switch‐like GTPase activity of MASL1 and thus part of a reciprocal regulatory relationship.

To address the intrinsic biases of these two approaches, we integrated the literature‐derived and experimentally derived data. The advantage of this strategy is that the microarray data will dilute the ascertainment bias of the literature‐derived network, while the literature‐derived network will supply the reproducibility element and aid prioritization of positive hits from the microarray experiments. By overlaying this data, numerous interactors common to both datasets became evident, including interactors from the same seed origin, reinforcing confidence in the protein microarray data, and interactors that were replicated between the two approaches but in association with different seeds of origin (Figure 4), opening new avenues for future functional investigation. These common nodes across both approaches were used to construct the common core network (Figure 4), which illustrates the potential overlap in ROCO protein interaction profiles.

The probability of proteins interacting within the cellular environment is subject to a number of important variables, including both temporal and spatial patterns of expression. Therefore, we subjected the interactors of the common core network to tissue‐specific gene expression profiling using data from GTEx (Table 3, Supporting Information). Although this represents a crude type of analysis (i.e., temporal expression and intracellular localization are not taken into account), it provided another way to assess the probability of the interactions reported in the common core network based on co‐existence of protein transcripts in human tissues. Particularly, we gathered that the highest frequency of co‐expressed interaction pairs was in the reproductive apparatus, followed by brain and intestine, whilst only skeletal muscle did not show any co‐expression. Additionally, ten proteins (ABL1, CALM1, CBLB, CDC42EP3, GAK, MRGBP, RPAP3, SNX9, STUB1, and TUBB4B) were co‐expressed with ROCO proteins in 12 out of the 13 tissues analyzed. This insight into tissue‐specific co‐expression supports the likelihood of the majority of pairwise interactions that have been reported in the literature and that have been assessed in a functional context.

To obtain functional insight into the ROCO protein interaction network, we performed functional enrichment analysis for the literature‐derived and common core networks, independently. The analysis of the former evidenced a diverse range of cellular functions (Figure 2A, Supporting Information), which support the concept of the ROCO proteins as hubs for a multitude of signaling cascades and hence challenging targets for therapeutic development.^4^ The analysis of the latter suggested a limited range of associated functional blocks: cell death, intracellular organization (particularly cell projections), protein metabolism, and response to stimulus (particularly stress response; Table 2 and Figure 2B, Supporting Information). In addition, functional enrichment analysis of individual ROCO protein interactomes indicated distinct functional themes for each seed protein (Table 2). In combination, these enrichment analyzes provide an overview of cellular functions associated with the ROCO proteins, suggesting potential convergent and divergent roles of these proteins within the cell, thus guiding future detailed assessments of ROCO protein function.

These analyzes provide a valuable foundation for understanding the ROCO protein interaction network. We here integrated peer‐reviewed literature, microarray, and co‐expression datasets to isolate common and distinct interactors of the ROCO proteins. We constructed a ROCO protein common core network highlighting the extent of commonality in the interaction profiles of these proteins. Using functional analysis approaches, we showed that, the ROCO proteins share a structurally conserved unit, which may be responsible for shared interactions (such as those with BAG2, CBLB, CDC42EP3, STUB1, and VGLL4) and as consequence, may influence the involvement of the ROCO proteins in common pathways identified (such as stress response and cell projection organization). However, despite this domain conservation, the ROCO proteins seem to have evolved largely divergent interactomes and associated functions within the cell (Table 2). This supports previous research into the functions of LRRK1 and LRRK2.^7^ The diversification of interactomes and biological functions of the ROCO proteins may reflect an evolutionary pressure toward phylogenetic differentiation of a single ancestral ROCO gene^51^ and may justify why the human ROCO proteins are differently associated with disease.

In summary, we utilized a confidence‐weighted data processing pipeline (WPPINA) to prioritize high‐throughput experimental results. Importantly, this approach provides the flexibility to incorporate data from a wide range of sources, and in the future could be further complemented by findings from yeast two‐hybrid and stable isotope labelling with amino acids in cell culture (SILAC) screens, for example. Together, this analysis highlights the value of a multi‐layered approach, combining bioinformatics with novel experimental data to better inform and accelerate laboratory investigations.

**Abbreviations**
DAPK1: death‐associated protein kinase 1
LRRK1: leucine‐rich repeat kinase 1
LRRK2: leucine‐rich repeat kinase 2
MASL1: malignant fibrous histiocytoma amplified sequence 1
WPPINA: weighted protein–protein interaction network analysis

## Conflict of Interest

The authors declare no conflict of interest.

## Supporting information

Supporting information

Supporting information

Supporting information

Supporting information

Supporting information

Supporting information

Supporting information

Supporting information

Supporting information

Supporting information

Supporting information

Supporting information
