# Supplementary material for: Comparative Protein Interaction Network Analysis Identifies Shared and Distinct Functions for the Human ROCO Proteins
Source: Proteomics. 2018 Apr 17;18(10):1700444. doi: 10.1002/pmic.201700444 (PMC5992104; doi:10.1002/pmic.201700444)
Supplement: Supplementary file 10 — Supporting information [file PMIC-18-na-s010.pdf]

PROTEOMICS

Supporting Information for Proteomics

DOI 10.1002/pmic.201700444

James E. Tomkins, Sybille Dihanich, Alexandra Beilina, Raffaele Ferrari, Nicolò Ilacqua, Mark R. Cookson, Patrick A. Lewis and Claudia Manzoni

Comparative Protein Interaction Network Analysis Identifies Shared and Distinct Functions for the Human ROCO Proteins

© 2018 The Authors. *Proteomics* Published by WILEY-VCH Verlag GmbH & Co. KGaA, Weinheim

g:Profiler (n=28)			Panther (n=17)			WebGestalt (n=10)		
**Functional block**	**Number of GO terms**	**% of enrichment**	**Functional block**	**Number of GO terms**	**% of enrichment**	**Functional block**	**Number of GO terms**	**% of enrichment**
cell death	5	17.9	cell death	3	17.6	cell death	2	20.0
cell cycle	0	0.0	cell cycle	3	17.6	cell cycle	0	0.0
development	0	0.0	development	1	5.9	development	0	0.0
enzyme	2	7.1	enzyme	1	5.9	enzyme	0	0.0
general	0	0.0	general	3	17.6	general	0	0.0
intracellular organisation	3	10.7	intracellular organisation	0	0.0	intracellular organisation	1	10.0
metabolism	0	0.0	metabolism	2	11.8	metabolism	2	20.0
protein metabolism	7	25.0	protein metabolism	0	0.0	protein metabolism	3	30.0
response to stimulus	11	39.3	response to stimulus	4	23.5	response to stimulus	2	20.0
