# Supplementary material for: Comparative Protein Interaction Network Analysis Identifies Shared and Distinct Functions for the Human ROCO Proteins
Source: Proteomics. 2018 Apr 17;18(10):1700444. doi: 10.1002/pmic.201700444 (PMC5992104; doi:10.1002/pmic.201700444)
Supplement: Supplementary file 12 — Supporting information [file PMIC-18-na-s012.docx]

**Supplementary Figures and Tables**

**
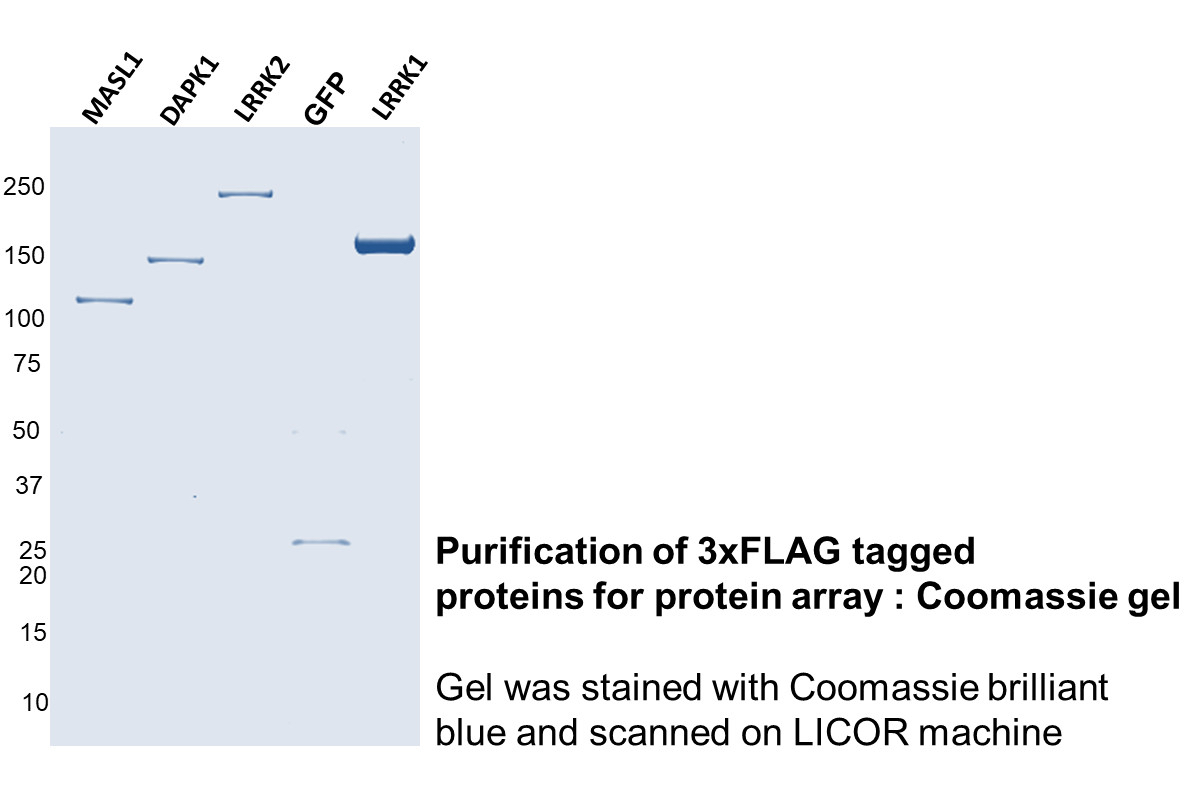
**

**Supplementary Figure S1 – Purification of 3xFLAG tagged proteins for protein microarray.** Gel stained with Coomassie brilliant blue and scanned on LICOR machine.

**(A)**


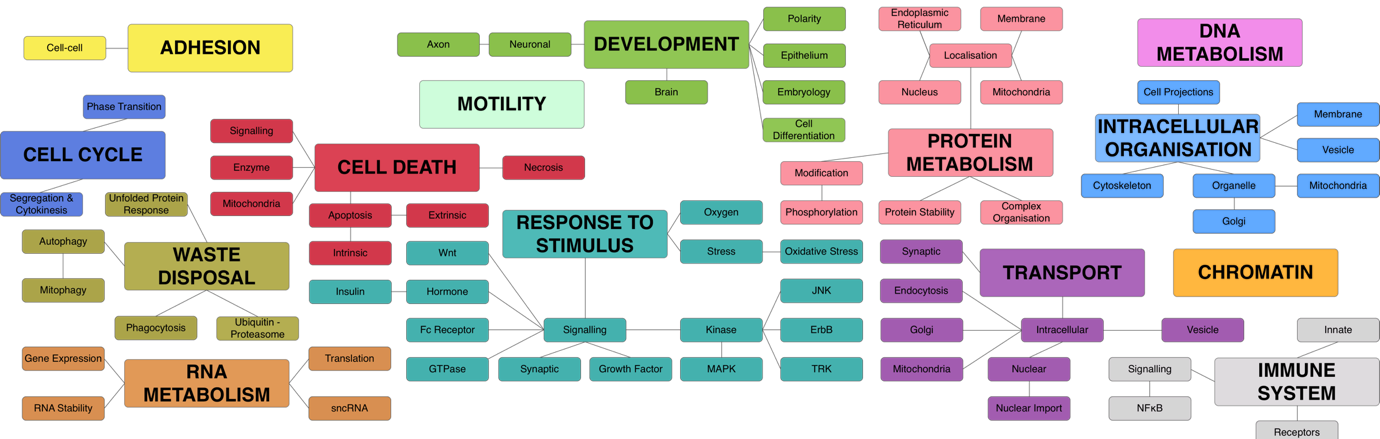


**(B)**


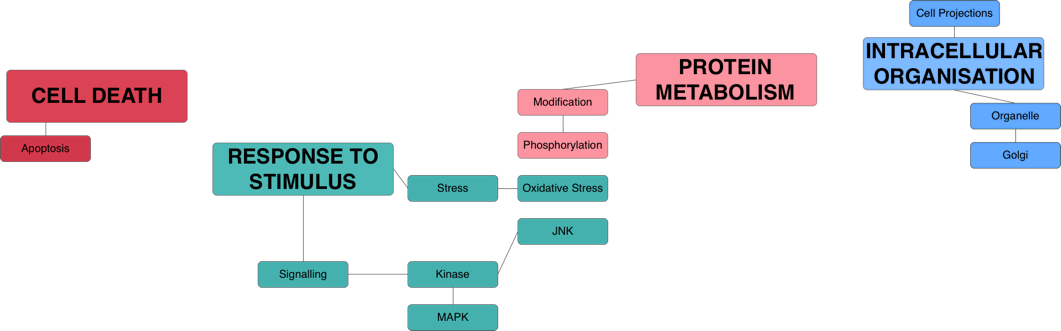


**Supplementary Figure S2** – **Overview of functional enrichment analysis for literature-derived network (A) and the common core network (B).** Gene ontology (GO) terms were grouped into functional blocks: adhesion, cell cycle, cell death, chromatin, development, DNA metabolism, enzyme, general, immune system, intracellular organisation, metabolism, motility, physiology, protein metabolism, response to stimulus, RNA metabolism, transport and waste disposal (Supplementary Files 4-9). GO terms that were categorised into the enzyme, general, metabolism or physiology functional blocks were discarded from the analysis, due to them providing limited and unspecific functional information. These 14 functional blocks were then subdivided into more specific semantic classes using a curated dictionary list to match GO terms to a custom grouped ontology, based on semantic similarity.

**Supplementary Table S1 - common interactors identified within the literature-derived network**

| **Seeds** | **Common interactor** | **Functional overview** | **References** |
| --- | --- | --- | --- |
| DAPK1 & LRRK2 | FADD (Q13158),  FAS-associated death  domain protein | Apoptosis, necrosis, autophagy, inflammation, innate immunity, cell proliferation, cell cycle progression, lipid metabolism | ^[1]^ ^[2]^ |
|  | MYO1B (O43795), Unconventional myosin-Ib | Intracellular transport, cell motility, cytoskeletal organisation | ^[3]^ |
|  | MYO1D (O94832), Unconventional myosin-Id | Intracellular transport, cell motility, cytoskeletal organisation | ^[3]^ |
| LRRK1 & LRRK2 | BAG5 (Q9UL15),  Bcl-2 associated athanogene 5 | Protein chaperone | ^[4]^ ^[5]^ |
|  | HSPA8 (P11142),  Heat shock cognate 71kDa protein | Protein chaperone,  transcriptional repression, autophagy | ^[6]^ |

[1] L. Tourneur, G. Chiocchia, *Trends Immunol.* **2010**, *31*, 260.

[2] H. Zhuang, X. Wang, D. Zha, Z. Gan, F. Cai, P. Du, Y. Yang, B. Yang, X. Zhang, C. Yao, Y. Zhou, C. Jiang, S. Guan, X. Zhang, J. Zhang, W. Jiang, Q. Hu, Z.-C. Hua, *EMBO Mol. Med.* **2016**, DOI 10.15252/emmm.201505924.

[3] M. A. Hartman, D. Finan, S. Sivaramakrishnan, J. A. Spudich, *Annu. Rev. Cell Dev. Biol.* **2011**, *27*, 133.

[4] M. Kabbage, M. B. Dickman, *Cell. Mol. Life Sci.* **2008**, *65*, 1390.

[5] X. Wang, J. Guo, E. Fei, Y. Mu, S. He, X. Che, J. Tan, K. Xia, Z. Zhang, G. Wang, B. Tang, *PLoS One* **2014**, *9*, e86276.

[6] F. Stricher, C. Macri, M. Ruff, S. Muller, *Autophagy* **2013**, *9*, 1937.

**Supplementary Table S2 – LRRK2 interactors identified by protein microarray experiment that would exceed the >2 score threshold if integrated into the WPPINA pipeline**

| **LRRK2 interactor** | | **Additional seed interaction** | |
| --- | --- | --- | --- |
| **Abbreviated name** | **Full protein name** | **Literature^+^** | **Protein microarray** |
| ANKS4B | Ankyrin repeat and SAM domain-containing protein 4B | - | MASL1 |
| BAG2 | BAG family molecular chaperone regulator 2 | DAPK1* LRRK1 | DAPK1 LRRK1 MASL1 |
| CDC42EP3 | Cdc42 effector protein 3 | LRRK1 | DAPK1 LRRK1 MASL1 |
| CUEDC1 | CUE domain-containing protein 1 | - | MASL1 |
| LGALS8 | Galectin-8 | - | - |
| NDUFAF7 | Protein arginine methyltransferase NDUFAF7, mitochondrial | - | - |
| ZRANB2 | Zinc-finger Ran-binding domain-containing protein 2 | - | - |

^+^unthresholded data

*interaction in thresholded literature-derived network

Full protein names as recommended by uniprot

**List of Supplementary Files**

**Supplementary Table S3 – Coexpression analysis of common core network interactors.** Shaded boxes indicate coexistence of interactor and seed protein mRNA in relation to a 3 RPKM expression threshold.

**Supplementary File S1 – Method reassignment ontology**

**Supplementary File S2 – List of positive hit interactors identified in the protein microarray screens**

**Supplementary File S3 – List of interactors in common core network**

**Supplementary File S4 – Literature-derived network g:Profiler functional enrichment analysis**

**Supplementary File S5 – DAPK1 interactome g:Profiler functional enrichment analysis**

**Supplementary File S6 – LRRK1 interactome g:Profiler functional enrichment analysis**

**Supplementary File S7 – LRRK2 interactome g:Profiler functional enrichment analysis**

**Supplementary File S8 – Common core network g:Profiler functional enrichment analysis**

**Supplementary File S9 – Functional block and semantic class contribution of each functional enrichment analysis**

**Supplementary File S10 – Common core network functional enrichment replication in g:Profiler, Panther and WebGestalt comparison**
